# Editorial: Early maternal and child health management and the impact of living environment

**DOI:** 10.3389/fpubh.2025.1626593

**Published:** 2025-06-17

**Authors:** Kathleen Baird, Hong Lu, Lihua Ren, Jiasi Yao, Wenjing Luo, Yinchu Hu

**Affiliations:** ^1^School of Nursing and Midwifery, Faculty of Health, University of Technology, Sydney, NSW, Australia; ^2^School of Nursing, Peking University, Beijing, China; ^3^School of Nursing, Hebei Medical University, Shijiazhuang, China

**Keywords:** maternal health, child health, healthy environments, pregnancy, child and family health

## Advancing maternal and newborn health: a call to action

Maternal and newborn health remains a fundamental pillar of global public health, reflecting broader imperatives of health equity, social justice, and sustainable development. As emphasized in the United Nations Sustainable Development Goals (SDGs) and particularly SDG 3, which aims to ensure healthy lives and promote wellbeing for all at all ages, improving outcomes during pregnancy, childbirth, and early childhood is not only a medical concern, but also a societal and ethical imperative. This special edition of *Frontiers in Public Health* brings together emerging research at the intersection of early maternal and child health management and the environmental contexts in which these lives begin and evolve. This edition comprises of 10 original research articles, one methods paper, one systematic review, and one study protocol. Collectively, these contributions reflect a broad thematic coherence centered on maternal and child health, while drawing from diverse geographic contexts including Africa, Asia, South America, and Europe.

## Maternal and child health in context

Growing evidence underscores the profound influence of environmental determinants—such as air and water quality, housing conditions, sanitation, and exposure to toxicants—on pregnancy outcomes and early childhood development. Despite increasing recognition, these environmental risks remain underrepresented in clinical protocols and public health strategies. This Research Topic addresses this critical gap, advocating for a more holistic, systems-based understanding of maternal and child health that acknowledges the intricate connections between ecological exposures and health trajectories. Articles in this submission which focus on this topic include.

## A global and multidisciplinary perspective

The articles featured in this Research Topic provide a rich and diverse examination of maternal and child health across continents—from the public hospitals of Ethiopia (Wondifraw et al.) to urban centers in China (Zhu et al.), Brazil (Lima-Soares et al.), and Malta (Battista et al.). They explore multifactorial influences on health outcomes, including biomedical, environmental, socioeconomic, and behavioral determinants. A consistent theme is the vulnerability of mothers and infants to systemic and environmental risks.

Studies investigating the impact of polycyclic aromatic hydrocarbons (Cheng et al.) and environmental tobacco smoke (Zhang et al.) on pregnant women and newborns reveal urgent concerns about prenatal environmental exposures and their metabolic consequences. Meanwhile, a mechanistic study (Jia et al.) on parental exposure to Nd_2_O_3_ demonstrates how intergenerational toxicity mediated through the brain-gut axis can impair neurodevelopment and cognition in offspring—adding new depth to our understanding of inherited environmental burdens.

## Barriers and innovations in care delivery

Access to timely and appropriate care is another crucial dimension. Research from East Africa (Terefe et al.) and Ethiopia (Wondifraw et al.) reveals systemic barriers to postnatal care and challenges in pediatric antiretroviral therapy adherence, calling for community-driven solutions and policy reform. In China, qualitative studies (Cai et al.) on kangaroo mother care provide insight into provider attitudes and implementation challenges, humanizing the complexities of neonatal care delivery in intensive care settings.

## Predictive models, preventive strategies, and mental health

Other studies focus on early risk detection and long-term health planning. These include predictive models for low birth weight in the context of gestational diabetes (Pan et al.), as well as the socioeconomic roots of birth outcomes and their link to peripubertal obesity (Lima-Soares et al.). Such findings reinforce the importance of health equity, social determinants, and proactive care models. A longitudinal analysis of perinatal outcomes in Malta (Battista et al.) over 15 years offers a historical perspective on progress and areas for improvement.

Notably, the Research Topic also highlights the mental health needs of obstetric and gynecological providers. As the demand for perinatal mental health screening grows, supporting healthcare professionals with knowledge, training, and systemic backing is essential for sustainable implementation.

## Toward an integrated, global approach

Together, these contributions form a compelling call for a multidisciplinary, evidence-based, and culturally responsive approach to maternal and child health. They underscore the urgency and global significance of this field, which lies at the intersection of medicine, public health, environmental science, and social policy. As maternal and child health remains a central focus of global health agendas, these studies highlight the need for integrated strategies that are adaptable across diverse settings and responsive to both emerging and persistent health inequities worldwide.

This Research Topic not only deepens our understanding of the early-life health landscape but also serves as a pivotal rallying point for continued research, collaboration, and innovation. The findings presented here underscore the pressing need for further investigation into the complex interplay of biomedical, environmental, and social factors that shape maternal and child health outcomes. We call on the global research and public health communities to expand upon this work, fostering interdisciplinary partnerships that explore the transgenerational impact of early-life health. By addressing persistent disparities, advancing evidence-based interventions, and understanding the long-term effects on future generations, we can ensure that every mother and child has the opportunity to thrive in a safe, supportive, and health-promoting environment. Ultimately, these efforts will contribute to the realization of global health goals and the wellbeing of future generations.

